# Kidney organoids for disease modeling

**DOI:** 10.18632/oncotarget.24438

**Published:** 2018-02-07

**Authors:** Elena Garreta, Nuria Montserrat, Juan Carlos Izpisua Belmonte

**Affiliations:** Gene Expression Laboratory, Salk Institute for Biological Studies, La Jolla, California, USA

**Keywords:** human pluripotent stem cells, kidney organoids, gene editing, CRISPR/Cas9, disease modeling

The kidney is formed during development by reciprocal interactions between the ureteric bud (UB) and the metanephric mesenchyme (MM), which promote the induction of nephron patterning and differentiation. Traditionally, UB and MM cells including nephron progenitor cells (NPCs) have been very difficult to isolate and maintain in culture due to their propensity to differentiate when outside their developmental niche. Remarkably, in recent years researchers have succeeded in prolonging the lifespan of mouse [1], rat [1], and human [2] NPCs *in vitro*, offering an avenue to expand the current knowledge of mammalian kidney development, and eventually for disease modelling and drug screening studies. Alternatively, renal progenitors have also been generated from human pluripotent stem cells (hPSCs) by mimicking early kidney developmental signals *in vitro*. Recently, different laboratories have been able to partially reproduce kidney organogenesis *in a dish* using hPSCs, successfully generating so-called kidney organoids [3,4,5,6]. Kidney organoids contain self-organized nephron-like structures composed of early podocyte cell clusters connected to tubular structures expressing markers of proximal tubules, loops of Henle and distal tubules [3,4,5,6]. In addition, kidney organoids display proximal tubular functionality *in vitro,* showing selective endocytosis of dextran cargoes [5,6], as well as responding to nephrotoxic agents [4,5,6].

Recent advances in the generation of kidney organoids *in vitro* together with state-of-art genome editing tools, including clustered regularly interspaced short palindromic repeats (CRISPR)/CRISPR-associated systems 9 (Cas9), represent a unique opportunity to study human kidney development and disease. Congenital kidney disorders can be modelled by introducing disease mutations in hPSCs and subsequently studying the disease phenotype in differentiated kidney organoids. Following this approach, Freedman and colleagues have recently reported an *in vitro* human model for polycystic kidney disease (PKD) by producing hPSCs with targeted biallelic mutations in the PKD1 and PKD2 genes encoding polycystin-1 (PC1) and polycystin-2 (PC2), respectively. CRISPR-mutant PKD and PKD2 knockout kidney organoids formed cysts in kidney tubules, recapitulating some characteristic features of this life-threatening renal disease *in vitro* [6]. In the same work, the authors also tested the function of hPSC lines that were gene edited so that they lacked the podocalyxin (PODXL) gene. PODXL-defective kidney organoids revealed junctional organization defects in podocyte-like cells, showing for the first time that hPSC-derived podocyte (hPSC-podocytes) cells could have the potential to recapitulate phenotypes of glomerulopathies *in vitro* [6]. In a follow up study it was shown that hPSC-podocytes inside kidney organoids exhibited a differentiation stage similar to mammalian podocytes at the capillary loop stage (CLS), and were able to establish junction-rich basal membranes and microvillus rich apical membranes [7]. In contrast, PODXL knockout hPSC-podocytes had defects in the assembly of microvilli, which resulted in failed junctional migration. Importantly, these defects were also found in CLS glomeruli of PODXL deficient mice, suggesting that PODXL has an essential role in mammalian podocyte maturation [7]. Although hPSC-podocytes could complete the process of junctional migration, they were unable to form bona fide foot processes characteristic of mature podocytes. In this regard, the current inability to produce vascularized kidney organoids able to sustain the development of glomerular capillary loops could be a major drawback for further podocyte maturation as well as kidney tissue growth.

Further improvements in kidney organoid methodologies are required to overcome major limitations, such as the lack of vascularization and the generation of a proper collecting duct system. Increasing experimental evidence have shown that the biophysical properties of the cell milieu significantly contributes to the modulation of stem cell differentiation, and ultimately to tissue formation and function. In this regard, Freedman and colleagues have recently shown that adherent forces play a critical role in increasing or decreasing cyst formation [8]. By culturing mutant kidney organoids in suspension instead of under adherent culture conditions, the authors were able to establish a highly efficient model of PKD cystogenesis, which phenotypically resembled PKD patient cysts [8]. They further showed that PKD2−/− organoids were defective in PC1 protein expression, while in PKD1−/− organoids PC2 expression levels were unchanged from isogenic controls. Indeed, knocking down PC2 protein in control hPSCs induced a decrease in PC1 protein. Altogether these findings indicated that PC2 was required for PC1 expression in human cells. Moreover, the authors proved that kidney organoid epithelia were able to remodel their extracellular matrix (ECM), and that this property was partially dependent on PC1, revealing a role of the ECM microenvironment in maintaining tubular shape and adhesion through interactions of PC1’s long extracellular domain [8]. These findings highlighted the importance of the ECM microenvironment in maintaining tubular architecture, playing an essential role at the earliest stages of PKD.

Overall, the above discussed experiments as well as others, demonstrate the power of CRISPR/Cas9 when combined with hPSCs derived organoids in order to explore the role of candidate genes related to kidney disease. Further knowledge in this area may open new venues for the systematic analysis of CRISPR/Cas9 edited patient mutations in kidney organoids, offering ideal platforms for drug and genetic screens aimed at identifying novel therapeutic targets.
